# The ad-libitum alcohol ‘taste test’: secondary analyses of potential confounds and construct validity

**DOI:** 10.1007/s00213-015-4171-z

**Published:** 2015-12-18

**Authors:** Andrew Jones, Emily Button, Abigail K. Rose, Eric Robinson, Paul Christiansen, Lisa Di Lemma, Matt Field

**Affiliations:** Psychological Sciences, University of Liverpool, Liverpool, L69 7ZA UK; UK Centre for Tobacco and Alcohol Studies, Liverpool, UK

**Keywords:** Ad-libitum, Alcohol, Awareness, Craving, Construct validity, Taste test

## Abstract

**Rationale:**

Motivation to drink alcohol can be measured in the laboratory using an ad-libitum ‘taste test’, in which participants rate the taste of alcoholic drinks whilst their intake is covertly monitored. Little is known about the construct validity of this paradigm.

**Objective:**

The objective of this study was to investigate variables that may compromise the validity of this paradigm and its construct validity.

**Methods:**

We re-analysed data from 12 studies from our laboratory that incorporated an ad-libitum taste test. We considered time of day and participants’ awareness of the purpose of the taste test as potential confounding variables. We examined whether gender, typical alcohol consumption, subjective craving, scores on the Alcohol Use Disorders Identification Test and perceived pleasantness of the drinks predicted ad-libitum consumption (construct validity).

**Results:**

We included 762 participants (462 female). Participant awareness and time of day were not related to ad-libitum alcohol consumption. Males drank significantly more alcohol than females (*p* < 0.001), and individual differences in typical alcohol consumption (*p* = 0.04), craving (*p* < 0.001) and perceived pleasantness of the drinks (*p* = 0.04) were all significant predictors of ad-libitum consumption.

**Conclusions:**

We found little evidence that time of day or participant awareness influenced alcohol consumption. The construct validity of the taste test was supported by relationships between ad-libitum consumption and typical alcohol consumption, craving and pleasantness ratings of the drinks. The ad-libitum taste test is a valid method for the assessment of alcohol intake in the laboratory.

## Introduction

Experimental investigations of the psychological processes that influence alcohol consumption are reliant on laboratory measures of alcohol-seeking. Measures include operant tasks, such as the progressive ratio task (Field et al. 2005; Van Dyke and Fillmore 2015), and the conceptually related alcohol purchase task, which measures how much people would be willing to pay for alcohol (MacKillop and Murphy 2007). The present study is focussed on another widely used measure, the ad-libitum taste test, which provides an unobtrusive and indirect measure of participants’ motivation to drink alcohol.

The ad-libitum taste test was first developed by Marlatt et al. (1973). Using a balanced placebo design, male alcoholics and controls were randomised to receive either alcohol or placebo, and they were informed that they were receiving either alcohol or placebo. They were asked to rate these beverages on a series of adjectives. The taste ratings concealed the genuine purpose of the taste test, which was to unobtrusively record how much of the available drink participants would consume. This paradigm or slight variations thereof has since become widely used in laboratory studies. Variations include the availability of a second beverage (usually a soft drink) in order to control for thirst and achieve consilience with animal paradigms such as the two-bottle free-choice procedure (Tabakoff and Hoffman 2000), and/or replacing the alcoholic beverage with a non-alcoholic alternative to mitigate the pharmacological effects of alcohol intoxication (Christiansen et al. 2013). The taste test has been used to investigate a number of potential influences on alcohol consumption, including impulse control (Christiansen et al. 2012; Jones et al. 2011), alcohol cues (Colby et al. 2004; Jones et al. 2013b; Van Dyke and Fillmore 2015), and social influences (Quigley and Collins 1999), and it has been used to establish initial proof of concept for novel behavioural interventions (Bowley et al. 2013; Field and Eastwood 2005; Jones and Field 2013).

Despite widespread use of the taste test, there has been no systematic investigation of its construct validity and variables that may compromise it (Leeman et al. 2010). For example, time of day and day of the week are known to influence alcohol consumption outside of the laboratory: people are more likely to drink alcohol on weekends (Friday, Saturday and Sunday) compared to midweek, and after 18:00 rather than earlier in the day (Kushnir and Cunningham 2014; Liang and Chikritzhs 2015). One implication is that participants’ willingness to consume alcohol in laboratory studies may be influenced by the time of the day and the day of the week, such that they are unwilling to drink alcohol at noon on Monday, which would compromise the validity of the taste test for participants who complete it at this time. Another potential confound is participants’ awareness that their consumption is being monitored. In a recent study involving an ad-libitum taste test with food (rather than alcohol), we demonstrated that participants who were aware that their intake was being monitored reduced their food consumption (Robinson et al. 2014). Furthermore, a subsequent meta-analysis demonstrated that participant awareness that food intake is being monitored may compromise construct validity in laboratory eating behaviour studies (Robinson et al. 2015). However, to date, the influence of participant awareness on ad-libitum consumption of alcohol has not been investigated.

Regarding construct validity, if individual differences in ad-libitum alcohol consumption during a taste test are predictive of drinking behaviour in naturalistic settings outside of the laboratory, we would expect to see significant positive correlations between the amount of alcohol that people voluntarily consume in the lab and their drinking behaviour outside of it. Two studies investigated this issue and reported some correspondence between the volume of alcohol consumed in the lab and self-reported drinking behaviour outside the lab, but both studies were underpowered to detect small associations, which may account for the inconsistent findings that were observed (Leeman et al. 2009, 2013).

In the current study, our primary aims were to investigate variables that may compromise the construct validity of the ad-libitum taste test and thoroughly investigate its construct validity. We conducted secondary analysis on data from studies conducted in our laboratory that incorporated an ad-libitum taste test. We investigated the relationships between alcohol consumption and time of day, day of the week and participant awareness. We used regression analyses to investigate the construct validity of the task by including participants’ gender, typical alcohol consumption, subjective craving, scores on the Alcohol Use Disorders Identification Test (AUDIT) and perceived pleasantness of the drinks as predictors of ad-libitum consumption. We hypothesised that participants would consume more alcohol later in the day, and that participants who were aware that their alcohol consumption was being monitored would consume less alcohol than those who were unaware. Finally, to confirm its construct validity, we predicted that pleasantness ratings of the drinks together with individual differences in retrospective alcohol consumption, subjective craving and scores on the Alcohol Use Disorders Identification Test would predict the volume of alcohol consumed during the taste test.

## Methods

We included data from previous studies conducted in our laboratory over the previous 10 years (2005–2015). We included all available studies that incorporated an ad-libitum taste test and from which we were able to infer the time of day and day of the week in which the testing session took place. Our analysis was limited to data from our own laboratory because participant-level time and date information are not reported in published manuscripts. We were able to obtain time and date information because the majority of our studies administered computerised tasks immediately before or after the taste test, so we were able to use time and date stamps in computer files to calculate the time and day that individual participants completed the taste test. Available data points were extracted from 12 independent studies, and details of each study are provided in Table 1. The aim in most of these studies was to investigate the influence of an experimental manipulation on alcohol consumption. In order to control for and examine whether the ad-libitum taste test was sensitive to the experimental manipulations used within each study, we created a condition variable and coded this based on the original hypotheses in each study (control group, condition expected to increase alcohol consumption and/or condition expected to reduce alcohol consumption). Two studies employed a within-subject design (Christiansen et al. 2013; Jones et al. 2013b), and in these cases, we used data from the control condition only, to ensure independence of data points. Selection of studies for inclusion and coding of data was performed and agreed by two authors (AJ and EB).

**Table 1:** Description of studies and variables included in the analyses

Study	Description of study and experimental groups	Measures included	Ad-libitum taste test
Di Lemma (in preparation) *N* = 120	Proof-of-concept behavioural intervention examining inhibitory control and approach bias training: Inhibition training (decreased expected) *N* = 30 Inhibition control (increased expected) *N* = 30 Avoid training (decreased expected) *N* = 30 Avoid control (increased expected) *N* = 30	AUDIT; awareness; day/time; alcohol cons.	2 × 200 ml (400 ml) of alcoholic beverages and 2 × 200 ml (400 ml) of soft drink.
Christiansen et al. (2013) *N* = 23	Acute alcohol intoxication study: Only control group was used as this was a within-subject design.	AUDIT; day/time; alcohol cons.	275 ml of non-alcoholic beer^a^ and 275 ml of soft drink.
Christiansen et al. (2012b) *N* = 80	Examining the effects of ‘ego-depletion’ on ad libitum alcohol consumption. Ego depletion group (increased expected) *N* = 40 Control (control) *N* = 40	AUDIT; craving (DAQ); day/time; alcohol cons.	3 × 255 ml of alcoholicbeverages.
Field et al. (2007) *N* = 60	Proof-of-concept behavioural intervention examining attentional bias training: Attend alcohol (increased expected) *N* = 20 Avoid alcohol (decreased expected) *N* = 20 Control (control) *N* = 20	AUDIT; craving (DAQ): day/time; pleasant; alcohol cons.	250 ml of alcoholic beverage and 250 ml of soft drink.
Jones et al. (2011) *N* = 90	Priming disinhibited mind-sets through instructions: Restraint (decreased expected) *N* = 31 Disinhibited (increased expected) *N* =30 Control (control) *N* = 29	AUDIT; awareness; craving (AAAQ); day/time; pleasant; alcohol cons.	275 ml of alcoholic beverage and 275 ml of soft drink.
Jones and Field (2013 experiment 1) *N* = 90	Proof-of-concept behavioural intervention examining inhibitory control training: Alcohol restraint (decreased expected) *N* = 30 Neutral restraint (control) *N* = 30 Disinhibition (increased expected) *N* = 30	AUDIT; awareness; craving (AAAQ); day/time; pleasant; alcohol cons.	250 ml of alcoholic beverage and 250 ml of soft drink.
Jones and Field (2013 experiment 2) *N* = 60	Proof-of-concept behavioural intervention examining inhibitory control training: Alcohol restraint (decreased expected) *N* = 30 Neutral restraint (control) *N* = 30	AUDIT; awareness; craving (AAAQ); day/time; pleasant; alcohol cons.	250 ml of alcoholic beverage and 250 ml of soft drink.
Jones et al. (2012) *N* = 14^b^	Examining the manipulation of beliefs on ad libitum consumption. High restraint beliefs (increased expected) *N* = 7 Low restraint beliefs (decreased expected) *N* = 7	AUDIT; awareness; craving (AAAQ); day/time; pleasant; alcohol cons.	250 ml of alcoholic beverage and 250 ml of soft drink.
Jones et al. (2013b) *N* = 60	Examining the effects of cue-reactivity on ad libitum consumption. Alcohol exposure (increased expected) *N* = 30 Water exposure (control) *N* = 30	AUDIT; awareness; craving (AAAQ); day/time; pleasant; alcohol cons.	250 ml of alcoholic beverage and 250 ml of soft drink.
Jones et al. (2013a) *N* = 16	Priming disinhibited mind-sets through instructions: only control group was used as this was a within-subject design.	AUDIT; craving (AAAQ); day/time; alcohol cons.	250 ml of alcoholic beverage and 250 ml of soft drink.
McGrath et al. (in preparation)^b^ *N* = 86	Examining the effects of acute stress on ad libitum alcohol consumption: Stress (increased expected) *N* = 43 Control (control) *N* = 43	AUDIT; awareness; craving (AAAQ); day/time; pleasant; alcohol cons.	3 × 300 ml (900 ml) of alcoholic beverages.
Robinson (unpublished data) *N* = 63	Examining the effects of participant awareness on ad-libitum consumption: Reduced awareness (increased expected) *N* = 20 Unaware (control) *N* = 22 Heightened awareness (decreased expected) *N* = 21	Day/time; pleasant; alcohol cons;	275 ml of alcoholic beverage and 275 ml of soft drink.

Groups were recoded for analyses based on hypothesised group differences in alcohol consumption (increased expected consumption, decreased expected consumption and control groups)

*AAAQ* Approach and Avoidance of Alcohol Questionnaire (inclined subscale), *Alcohol cons* units of alcohol consumed in the previous week, *AUDIT* Alcohol Use Disorders Identification Task, *Awareness* participants answered a multiple choice question examining if they were aware of the aims of the taste test, *DAQ* Desire for Alcohol questionnaire (mild craving subscale), *Pleasant* ratings of ‘pleasantness’ of the alcoholic beverage during the taste-test

^a^Non-alcoholic beer was used in this study. Pilot studies from our lab demonstrated that participants believe the beverage to be alcoholic

^b^Not full sample from publication, data from time of day were lost due to computer error

### Participants

In all studies, participants were non-dependent social drinkers and were predominantly university students (although occupational status was not consistently recorded). Participants in all studies were recruited if they consumed at least one unit of alcohol per week, whereas some studies recruited only ‘heavy drinkers’, defined as those who consumed alcohol in excess of UK government guidelines for safe drinking (Edwards 1996), which is ≥14 units per week for females and ≥21 units for males. Furthermore, all participants had to report regular consumption or liking of the type of beverages that were to be offered during the taste test, e.g. beer. Previous or current diagnosis of alcohol or other substance use disorder was always an exclusion criterion. We verified participants’ abstinence from alcohol by taking a breathalyser reading at the beginning of the studies; any participants with a breath alcohol level above zero were not permitted to take part. In all studies, prior to taking part, participants were told that alcohol may be available, and that they should not drive or operate heavy machinery for the remainder of the day. All participants provided informed consent, and each study was approved by the University of Liverpool’s committee for research ethics.

### Measures

#### Ad-libitum taste test

Different variants of the taste test were used in different studies, as detailed in Table 1. Most studies (*n* = 10) required participants to taste both alcohol and non-alcoholic (soft) drinks, whereas a smaller number required participants to taste different types of alcoholic drinks (*n* = 2). During the ad-libitum sessions, all drinks were provided at the same time (rather than consecutively). Participants were asked to rate drinks on different gustatory dimensions, e.g. gassy, bitter, and were explicitly asked to ‘drink as much or as little as you like in order to make accurate judgments’. Any identifying information of the beverages (brands, labels) was always removed. After participants had finished rating the drinks, the drinks were removed from the laboratory and measured *after* participants had been discharged from the study. The true nature of the taste test was always obscured with a cover story; for example, participants were informed that the study investigated the relationship between cognitive performance and taste perception of different drinks.

In order to ensure comparability across studies, we computed alcohol consumed as a percentage of the total alcohol that was available during the taste test, and this served as the primary dependent variable in all analyses. On average, participants consumed 34.61 % (±26.41) of the available alcohol during the taste tests.

### Indicators of construct validity

#### Typical alcohol consumption: timeline follow back drinking diary (Sobell and Sobell 1992)

Participants’ typical weekly alcohol consumption was assessed with a retrospective diary, the timeline follow back (TLFB). The TLFB has acceptable reliability in both dependent and non-dependent populations (Cohen and Vinson 1995; Hoeppner et al. 2010). The majority of studies required participants to record their alcohol consumption (in UK units) over the previous 2 weeks, although two studies recorded alcohol consumption over 1 week (consumption over 1 week tends to be highly correlated with consumption over 2 weeks (Vakili et al. 2008)). The volume of alcohol consumed per week, in UK units, was the variable used.

#### Alcohol Use Disorders Identification Task (Babor et al. 2001)

The Alcohol Use Disorders Identification Task (AUDIT) is a paper-and-pencil measure of hazardous drinking. It is a 10-item scale with each item scored 0–4. According to the WHO guidelines, scores >8 are indicative of hazardous or harmful use, with a risk of dependence. The AUDIT has a high degree of internal consistency and adequate test-retest reliability (Reinert and Allen 2007).

#### Craving

Craving was measured using one of the two craving scales: the Approach and Avoidance of Alcohol Questionnaire, ‘right now’ version (AAAQ (McEvoy et al. 2004)), or the Desire for Alcohol Questionnaire (DAQ (Love et al. 1998)). We included the inclined subscale from the AAAQ and the mild desires and intentions from the DAQ, both of which capture momentary inclinations to drink alcohol (rather than uncontrollable desires or other aspects of subjective craving). Subscales were standardised as *z* scores to ensure comparability across studies. If craving was measured more than once during the experiment, we took the measure closest in time before participants completed the taste test, as this would not be contaminated by the acute effects of alcohol (Rose and Duka 2006).

#### Pleasantness

In several studies, participants were asked to rate dimensions of the drinks on visual analogue or likert scales. We included participants’ ratings of the ‘pleasantness’ of the alcoholic drinks.

### Confounds

#### Time and day of the week

We coded time of day by examining time and date stamps from computerised tasks and used these to estimate the time that participants began the taste test. For ethical and practical reasons, all studies took place after 12:00 pm. We coded time of day as a continuous variable expressed as minutes after 12:00 pm when participants initiated the taste test. Day of the week was coded nominally (Monday, Tuesday, Wednesday, Thursday, and Friday).

#### Awareness

Seven studies (total of 520 participants; 213 males, 307 females) provided a funnelled debrief to participants to assess their awareness of the aims of the study and the measures that were administered. Participants’ awareness of the purpose of the taste test was assessed with the following multiple choice question ‘The purpose of the taste test was to…’. Of the 3–5 possible answers, the correct answer was ‘to measure how much alcohol I drank’. Participant awareness was coded dichotomously (1 = aware or 0 = unaware).

### Statistical power calculation

We obtained two correlations from previous research (Leeman et al. 2009) between ad-libitum consumption and craving (*r* = 0.32) and typical consumption (*r* = 0.21). A power calculation conducted in G*Power demonstrated that using the smaller and more conservative of the two correlations, a sample size of at least 241 would be required to find an association with *α* level = 0.05 and estimated power of 0.95. We also calculated that 178 participants would be needed to find a small to medium effect size for a multiple regression with 10 predictors at *α* = 0.05 and estimated power of 0.95. Therefore, all subsequent analyses were more than adequately powered.

## Results

### Participant characteristics (see Table 2)

**Table 2:** Baseline characteristics of variables included in the analyses, split by gender

	Male	Female
Alcohol cons.	30.53 (16.67)	21.95 (11.71)
AUDIT	14.41 (4.97)	13.95 (5.2)
Craving^a^	0.13 (0.97)	−0.12 (1.00)
Pleasantness	6.26 (2.17)	5.46 (5.83)
Time of day^b^	186.65 (104.12)	189.35 (106.86)

Values are means (±SDs)

^a^Craving scores standardised for each study (*z* scores)

^b^Minutes after midday in which ad-libitum session began

We obtained data from a total of 762 participants (300 male, 462 female), with a mean age of 20.82 ± 3.10 years. One participant was removed from analysis because his weekly alcohol consumption was an outlier (>115 units). Independent sample *t* tests indicated that males consumed significantly more alcohol per week than females (*t*(759) = 4.89, *p* < 0.001, *d* = 0.36), and they reported higher craving prior to the taste test (*t*(554) = 2.94, *p* < 0.01, *d* = 0.25), but there were no gender differences in AUDIT scores (*t*(697) = 1.18, *p* = 0.24).

*Time and day*: Ad-libitum sessions began between 12:17 and 20:20 pm. Time of day, measured as minutes after midday, was not significantly associated with volume of alcohol consumed during the taste test (*r* = 0.059, *p* = 0.10). Day of the week measured using a one-way ANOVA (Monday–Friday) was also not associated with volume of alcohol consumed during the taste test (F (4752) = 1.71, *p* = 0.14). There were no significant correlations between time of day and alcohol consumption when analysed separately across days of the week (rs < 0.10 ps > 0.18).

*Participant awareness*: Overall, 35.80 % of participants guessed the awareness of the taste test. However, participant awareness was not associated with the volume of alcohol consumed during the taste test (*t*(518) = 0.35, *p* = 0.72). The addition of gender did not moderate this effect (*p* = 0.54).

*Construct validity* (Table 3): We performed block adjusted multiple linear regression analysis to investigate predictors of alcohol consumption during the taste test. All collinearity diagnostics were in the tolerable range (VIFs < 1.39). The final model was significant and predicted 23 % variance in ad-libitum consumption (*R*^2^ = 0.23; F (9387) = 11.51, *p* < 0.01). Both participant gender and experimental condition were significant predictors of alcohol consumption. Most importantly, after controlling for participant age, gender and experimental condition, we found that weekly alcohol consumption, craving and pleasantness ratings all emerged as significant predictors of alcohol consumption during the taste test (see Table 3). Awareness or time of day did not significantly predict ad-libitum consumption in the model.

**Table 3:** Multiple hierarchical linear regressions investigating construct validity of the ad-libitum taste test

	Cumulative model		Individual predictors	
	*R* ^2^ change	*F* change	*Β* (SE)	95 % CI
Step 1	0.17	19.50*		
Age			0.46 (0.37)	−0.27–1.20
Gender			13.54 (2.48)*	8.66–18.42
Cond1			8.36 (2.62)*	3.24–13.48
Cond2			2.37 (3.04)	−3.61–8.36
Step 2	0.07	5.42*		
AUDIT			−0.46 (0.24)	−0.93–0.22
Alcohol cons			0.17 (0.08)**	0.09–0.33
Craving			4.54 (1.24)*	2.12–6.97
Pleasantness ratings			1.02 (0.49)**	0.49–1.99
Time of day			0.01 (0.01)	−0.02–0.03
Awareness			0.18 (2.51)	−4.76–5.11

Dependent variable: percentage of alcohol consumed of total alcohol available

Cond1: dummy coded (‘condition expected to increase alcohol consumption’ vs. control); Cond2: dummy coded (‘condition expected to reduce alcohol consumption’ vs. control)

*p < 0.01; **p < 0.05

## Conclusions

Our secondary analysis of data from studies that included an ad-libitum alcohol taste test demonstrated that participants’ alcohol consumption during the test is not influenced by time of day or day of the week, or their awareness that alcohol consumption is being monitored. Most importantly, we obtained evidence for the construct validity of the taste test: ad-libitum consumption was sensitive to experimental manipulations designed to increase consumption and was predicted by participant gender, their typical alcohol consumption, subjective craving and the perceived pleasantness of the alcoholic drinks that were offered.

These findings confirm that the ad-libitum taste test is a valid and sensitive instrument for the assessment of alcohol consumption in laboratory studies. Furthermore, findings support previous claims that participants’ alcohol consumption in the laboratory is representative of their drinking behaviour outside of the lab (Leeman et al. 2009, 2013). However, individual differences in scores on the AUDIT were unrelated to alcohol consumption during the taste test, which suggests that alcohol consumption in the laboratory may not correspond to hazardous drinking per se.

Importantly, and contrary to our expectations, alcohol consumption during the taste test was unaffected by the time of the day or day of the week on which the testing session took place. Even though these variables clearly influence drinking behaviour outside of the laboratory (Liang and Chikritzhs 2015), they do not appear to confound participants’ behaviour in the laboratory. We speculate that this may be due to increased variability in the onset of drinking episodes in students, compared to the general population (Del Boca et al. 2004). It is also likely that outcome expectancies and drinking motives that underlie drinking behaviour tend to fluctuate over time outside of the laboratory (Dvorak et al. 2014; Monk and Heim 2014), but they are suppressed and remain relatively stable in the lab (see Wall et al. 2000). Future research should investigate the relationship between outcome expectancies, drinking motives and ad-libitum consumption in the lab.

Participants’ awareness of the purpose of the taste test also did not influence their alcohol consumption, a finding that does not correspond with findings from the food literature, which demonstrate that participants eat less if they know that their food intake is being monitored (Robinson et al. 2014). One explanation for this discrepancy is that the majority of the food studies examining awareness of observation involved young adult female participants who were offered high-calorie foods (Robinson et al. 2015), the consumption of which may be stigmatised in this population (Vartanian et al. 2007). In contrast, alcohol consumption may be seen as socially acceptable or desirable behaviour in young people (Pavis et al. 1997) and is not stigmatised in a similar way.

Our analysis has some limitations. First, we calculated alcohol consumption as the amount that participants consumed as a proportion of the total amount of alcohol available during the taste test. This was necessary given the heterogeneity across studies in terms of volume of alcohol that was offered, and the availability of alternative (non-alcoholic) drinks. These differences between studies may influence consumption during the taste test, because increased choice can increase consumption of foods and beverages (Hardman et al. 2015; Reibstein et al. 1975). Second, our ad-libitum sessions took place during the afternoon and early evening on weekdays, so we cannot rule out the possibility that time and day would have influenced alcohol consumption if testing had taken place in the mornings, evenings and/or at weekends. Related to this point, the construct validity of the taste test might be improved if testing sessions take place later in the evening (Liang and Chikritzhs 2015; Larsen et al. 2012), although this speculation awaits empirical testing. Third, the majority of the taste tests analysed offered beer as the alcoholic beverage alongside a soft drink. Even though liking for beer was an inclusion criterion for all of the studies, it may not have been participants’ *preferred* drink. There may also be an important gender difference in this regard. Beer is the most popular alcoholic drink in the UK for males, but not females (Office for National Statistics 2012), yet the majority of participants included in our analysis were female. Therefore, future studies that use the taste test could consider offering participants their preferred drink(s), or a range of different drinks, in order to ensure better matching between the alcoholic drinks offered during the taste test and those that participants typically consume. It is also important to investigate if the availability of a soft drink and the total amount of alcohol available influence the amount of alcohol consumed or moderate the effect of experimental manipulations on alcohol consumption (we were unable to consider these factors in the analyses reported here because of limited variability in methods used).

Future research should set out to identify other factors that may influence alcohol consumption during the ad-libitum taste test. Despite inclusion of multiple candidate variables, our analysis was only able to account for a relatively modest amount of total variance in alcohol consumption (23 %). We can speculate on potential confounds that may influence alcohol consumption such as glass shape (Attwood et al. 2012; Troy et al. 2015), type of alcohol available (Quigley and Collins 1999), and availability of soft drink alternatives (as discussed previously). The gender of the experimenter and concordance between participant and experimenter gender may also be important, but we could not investigate this issue here because the majority of researchers were male. Furthermore, construct validity may have been compromised by participants’ poor recall or deliberate under-reporting of their typical alcohol consumption (Monk et al. 2015). Nevertheless, we echo calls by Leeman et al. (2013) for authors to report correlations between ad-libitum alcohol consumption in the lab and their typical drinking behaviour, in the future studies. Future research should also examine whether the ad-libitum taste test has predictive validity for future alcohol consumption, for example, using real-time reporting via electronic devices (see Monk et al. 2015) or biochemical measures such as breath alcohol content (Glindemann et al. 2007). However, we note that drinking behaviour is generally consistent over time (Rueger et al. 2012), and retrospective drinking diaries yield accurate, reliable and fine-grained information about individual differences in alcohol consumption (Hoeppner et al. 2010).

To conclude, we provide evidence for the construct validity of the alcohol ad-libitum taste test as a measure of alcohol consumption in the laboratory. We found no evidence that time of day, day of the week or participants’ awareness that their alcohol consumption was being monitored had an effect on their drinking behaviour.
